# Supplementary material for: Integrated analysis of the aqueous humor microbiome and lens capsule transcriptome in high myopia cataract: a pilot study
Source: Front Med (Lausanne). 2026 Jun 16;13:1845205. doi: 10.3389/fmed.2026.1845205 (PMC13314463; doi:10.3389/fmed.2026.1845205)
Supplement: Supplementary file 3 [file Data_Sheet_1.zip › 6.PICRUSt2/Meta_Statistic/C372089/Wilcoxon/COG_abundance.Wilcoxon.diff.top30.heatmap.pdf]

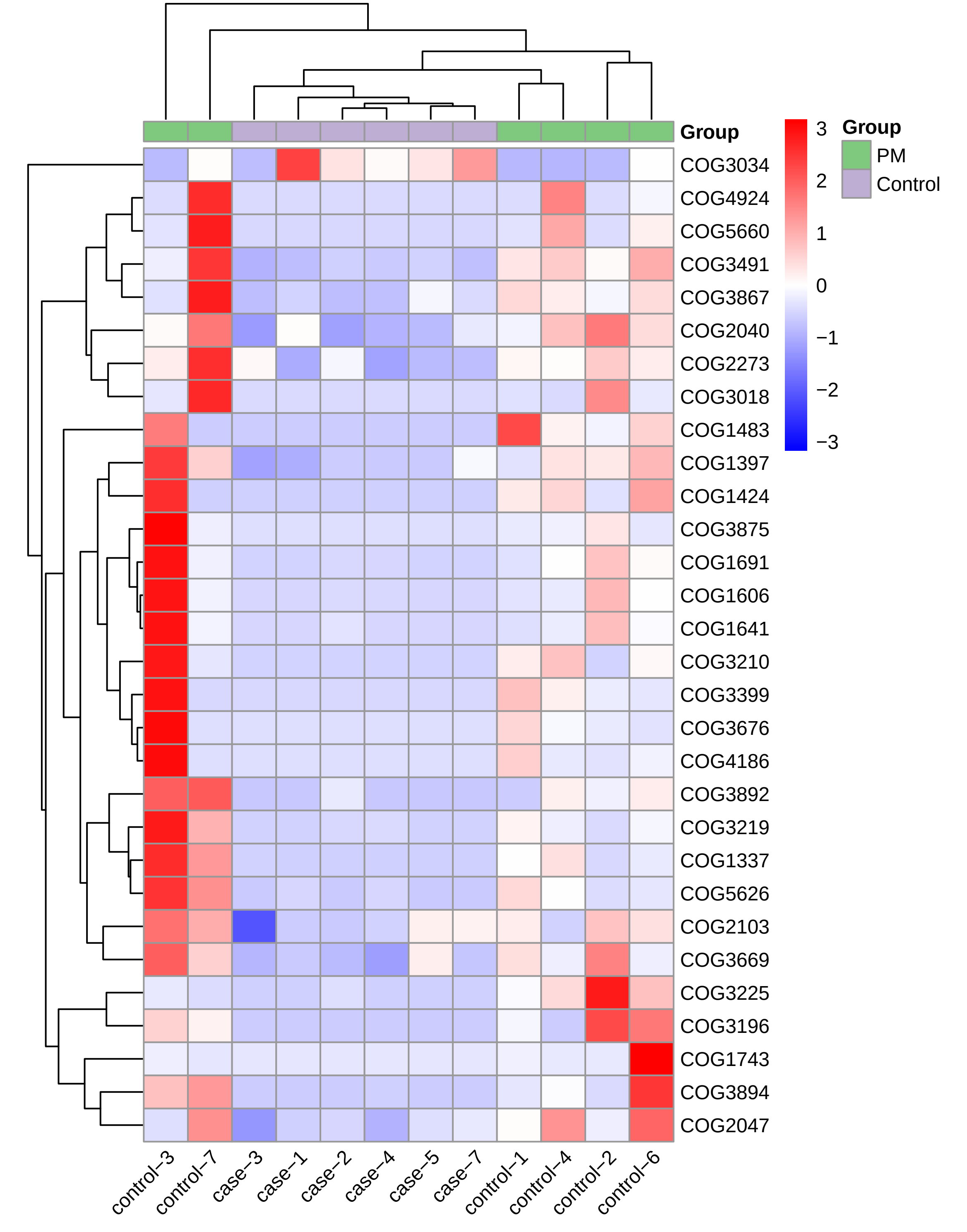
Group
COG3034
COG4924
COG5660
COG3491
COG3867
COG2040
COG2273
COG3018
COG1483
COG1397
COG1424
COG3875
COG1691
COG1606
COG1641
COG3210
COG3399
COG3676
COG4186
COG3892
COG3219
COG1337
COG5626
COG2103
COG3669
COG3225
COG3196
COG1743
COG3894
COG2047
control−3
control−7
case−3
case−1
case−2
case−4
case−5
case−7
control−1
control−4
control−2
control−6
3
2
1
0
−1
−2
−3
Group
PM
Control
